# Dietary Regulation of the Gut Microbiota Engineered by a Minimal Defined Bacterial Consortium

**DOI:** 10.1371/journal.pone.0155620

**Published:** 2016-05-13

**Authors:** Ting-Chin David Shen, Christel Chehoud, Josephine Ni, Evelyn Hsu, Ying-Yu Chen, Aubrey Bailey, Alice Laughlin, Kyle Bittinger, Frederic D Bushman, Gary D Wu

**Affiliations:** 1 Division of Gastroenterology, Perelman School of Medicine, University of Pennsylvania, Philadelphia, Pennsylvania, United States of America; 2 Department of Microbiology, Perelman School of Medicine, University of Pennsylvania, Philadelphia, Pennsylvania, United States of America; 3 Division of Gastroenterology and Hepatology, Department of Pediatrics, University of Washington School of Medicine, Seattle, Washington, United States of America; University of North Carolina at Chapel Hill, UNITED STATES

## Abstract

We have recently reported that Altered Schaedler Flora (ASF) can be used to durably engineer the gut microbiota to reduce ammonia production as an effective modality to reduce morbidity and mortality in the setting of liver injury. Here we investigated the effects of a low protein diet on ASF colonization and its ability to engineer the microbiota. Initially, ASF inoculation was similar between mice fed a normal protein diet or low protein diet, but the outgrowth of gut microbiota differed over the ensuing month. Notable was the inability of the dominant *Parabacteroides* ASF taxon to exclude other taxa belonging to the Bacteroidetes phylum in the setting of a low protein diet. Instead, a poorly classified yet highly represented Bacteroidetes family, S24-7, returned within 4 weeks of inoculation in mice fed a low protein diet, demonstrating a reduction in ASF resilience in response to dietary stress. Nevertheless, fecal ammonia levels remained significantly lower than those observed in mice on the same low protein diet that received a transplant of normal feces. No deleterious effects were observed in host physiology due to ASF inoculation into mice on a low protein diet. In total, these results demonstrate that low protein diet can have a pronounced effect on engineering the gut microbiota but modulation of ammonia is preserved.

## Introduction

The gut microbiota responds to multiple environmental stressors such as diet [1–4], antibiotic use [5], inflammation of the intestinal tract [6], and infection of the host with enteric pathogens [7]. By studying the gut microbiota in pediatric patients with Crohn’s disease, we have recently shown that the effects of these factors may be independent even if present simultaneously [8]. Amongst these, the impact of diet has received considerable attention as a potential modifiable factor that shapes the composition and/or function of the gut microbiota to prevent and/or treat disease [9]. The high-level efficacy of fecal microbiota transplantation (FMT) in the treatment of *Clostridium difficile* infections (CDI) is proof of concept that inoculating a host with a consortium of microbes has a meaningful effect on the composition of the gut microbiota [10]. The use of feces could be considered an untargeted approach with potential risks [11], but growing evidence suggests that the use of defined microbial consortia could be developed to treat disease [12, 13]. We have recently shown that the gut microbiota can be durably reconfigured to reduce fecal urease activity and ammonia production through oral inoculation of Altered Schaedler Flora (ASF), a defined microbial consortium that contains minimal urease gene content [13]. ASF comprises eight murine gut commensal bacterial strains (**S1 Table**) assembled in the 1970s and standardized by the National Cancer Institute in 1978 [14–16]. It is now commonly used to create gnotobiotic mice and/or to enhance the health of immunodeficient mouse strains.

Examples of co-metabolism between the gut microbiota and its mammalian host requiring host-derived substances include bile acids, mucous, and urea. The latter is particularly important for nitrogen flux between the host and the gut microbiota [17, 18]. As the primary source of nitrogen, dietary protein is essential to the synthesis of nucleic acids, amino acids, and other nitrogenous compounds. The catabolism of dietary protein by the host leads to hepatic formation of urea, a nitrogenous waste product that is excreted through the urine or delivered into the colon, where hydrolysis by bacterial urease results in the production of carbon dioxide and ammonia. Ammonia is a shared substrate for the synthesis of proteins, amino acids, and other small molecules by both the host and its microbiota. Although generally thought to be nutritionally beneficial to the host by enhancing nitrogen recycling, the production of ammonia by the gut microbiota can have deleterious effects in the setting of altered hepatic function, resulting in the development of neurotoxicity [19–21]. Under such conditions, a low protein diet (LPD) can be used to reduce systemic ammonia levels [22, 23].

By inoculating mice with ASF after the endogenous microbiota has been reduced through the use of antibiotics and polyethylene glycol (PEG), the composition of the gut microbiota can be durably modified in composition as well as function. Functionally, there was a long-lasting reduction in fecal ammonia that was effective in reducing morbidity and mortality in the thioacetamide model of liver injury [13]. Since (1) the absorption of fecal ammonia produced by the gut microbiota may be an important source of nitrogen for the host especially in the setting of dietary protein restriction [17], and (2) low protein diets are used clinically in patients with hyperammonemic inborn errors of metabolism [24], there are a number of questions about the impact of diet on the engineering of the gut microbiota to reduce ammonia production. What is the effect of a LPD on the ability of a defined bacterial consortium to colonize in the gut? Does a LPD have an effect on the composition of the engineered microbiota? Will the ammonia reduction by microbiota engineering be sustained and exhibit lower levels than those achievable by a LPD alone? And lastly, will a significant reduction in gut microbiota ammonia production be deleterious to the host on a LPD?

Here, we address these questions by inoculating mice on a LPD with either feces from conventionally-reared mice (Normal Feces, or NF) or with ASF, monitoring the resultant composition of the gut microbiota over time by 16S tagged sequencing, assessing functionality by quantifying fecal ammonia levels, and investigating the impact on the host by metabolic profiling. Although a LPD has no effect on the ability of ASF to colonize the gut of the host upon inoculation, the resultant engineered state of the microbiota is altered primarily due to the re-emergence of S24-7, a specific bacterial taxonomic family within the Bacteroidetes phylum. Despite this alteration, fecal ammonia levels remain diminished and without consequence to the metabolic physiology of the host on a LPD.

## Results

### LPD impacts host physiology and nitrogen metabolism but modestly alters the composition of the gut microbiota

**Fig 1:**
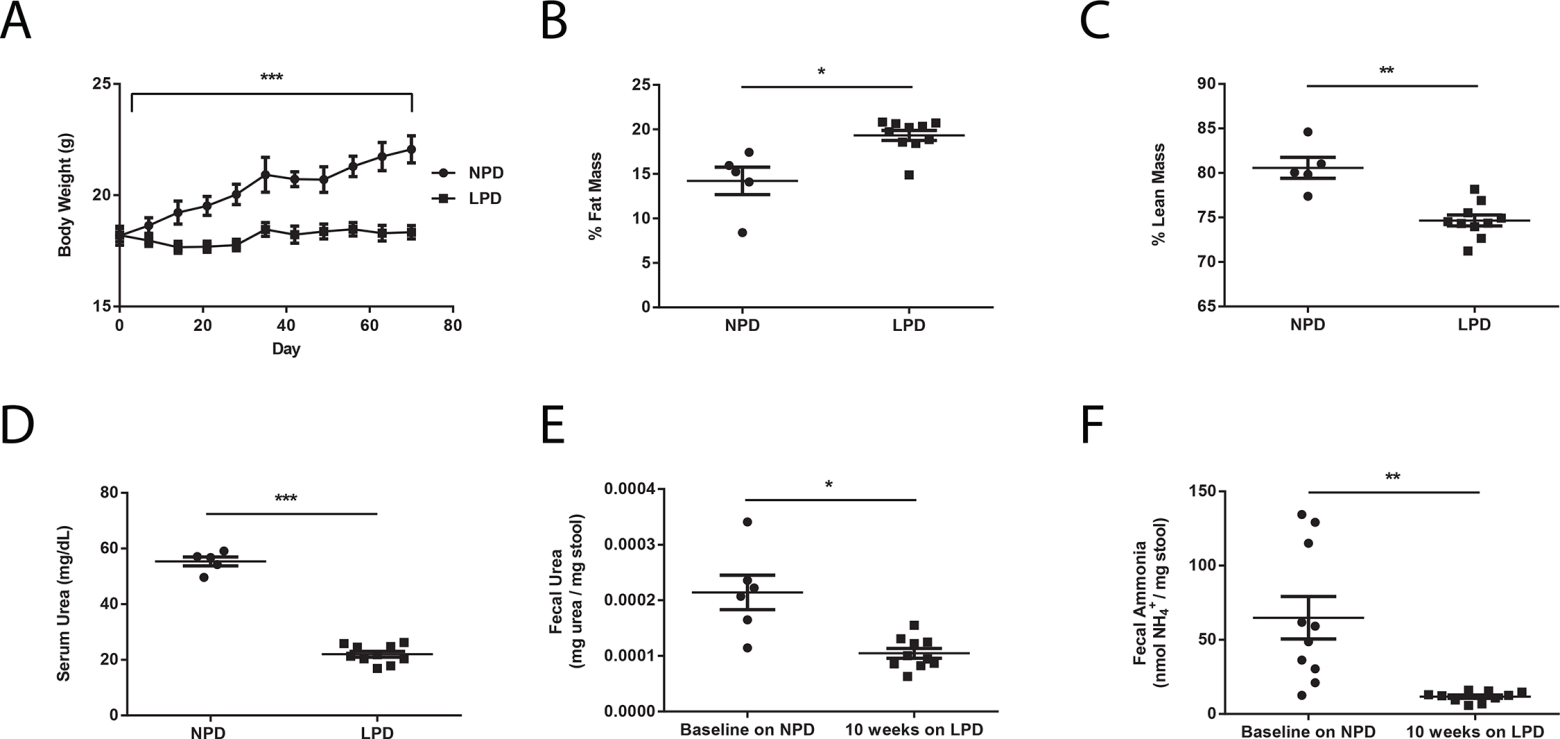
Changes in murine physiology and nitrogen metabolism on a LPD. Differences in (A) body weight (n = 5 in NPD group, n = 10 in LPD group), (B) % fat mass, (C) % lean mass, and (D) serum urea concentrations between NPD-fed and LPD-fed mice. (E) Fecal urea and (F) fecal ammonia levels at baseline on the NPD and after placement on the LPD. Values represent mean ± SEM. Statistical significance in body weight determined by two-way ANOVA with repeated measures; statistical significance in other parameters determined by paired and unpaired two-tailed Student’s t test. *p<0.05, **p<0.01, ***p<0.001.

We first set out to investigate the effects of a LPD on both the murine host and the gut microbiota. Fifteen adult female C57BL6J mice were placed on an open source irradiated purified rodent diet containing normal amount of dietary protein at 21% by kilocalories (AIN-76A), henceforth referred to as normal protein diet (NPD, **Table 1**) for one week upon arrival into the University of Pennsylvania SPF vivarium. Subsequently, ten of the 15 mice were switched to an irradiated low protein diet (LPD, **Table 1**) formulated using AIN-76A as the base that contains 3% protein by kilocalories. The LPD was made isocaloric by proportionally increasing carbohydrate content while keeping fat content unchanged. The remaining five mice continued to be fed the NPD. We monitored physiological changes in these mice using body weight and food intake measurements as well as body composition determination via nuclear magnetic resonance (NMR) imaging. We found that compared to NPD-fed mice, LPD-fed mice exhibited poor weight gain despite equivalent caloric consumption (**Fig 1A**). LPD-fed mice also demonstrated increased fat mass and decreased lean mass (**Fig 1B and 1C**). Corresponding to a reduction in serum urea concentration compared to NPD-fed mice (**Fig 1D**), LPD-fed mice exhibited significant reductions in fecal urea and fecal ammonia levels after ten weeks on the LPD (**Fig 1E and 1F**). These results are consistent with the fundamental role that dietary protein plays in host nitrogen balance. Reduction in dietary protein may have an effect on the gut microbiota by reducing the delivery of urea to the colonic environment leading to the reduction in fecal ammonia levels.

**Table 1:** Components of normal protein diet (NPD) and low protein diet (LPD).

	NPD		LPD	
	gram%	kcal%	gram%	kcal%
Protein	20	21	3	3
Carbohydrate	66	68	83	85
Fat	5	12	5	12
Total		100		100
kcal/gram	3.9		3.9	
**Ingredient**	**gram**	**kcal**	**gram**	**kcal**
Casein	200	800	30	120
DL-Methionine	3	12	0.45	1.8
Corn Starch	150	600	190	760
Sucrose	500	2000	632.5	2530
Cellulose, BW200	50	0	50	0
Corn oil	50	450	50	450
Mineral Mix S10001	35	0	35	0
Vitamin Mix V10001	10	40	10	40
Choline Bitartrate	2	0	2	0
**Total**	**1000**	**3902**	**1000**	**3902**

**Fig 2:**
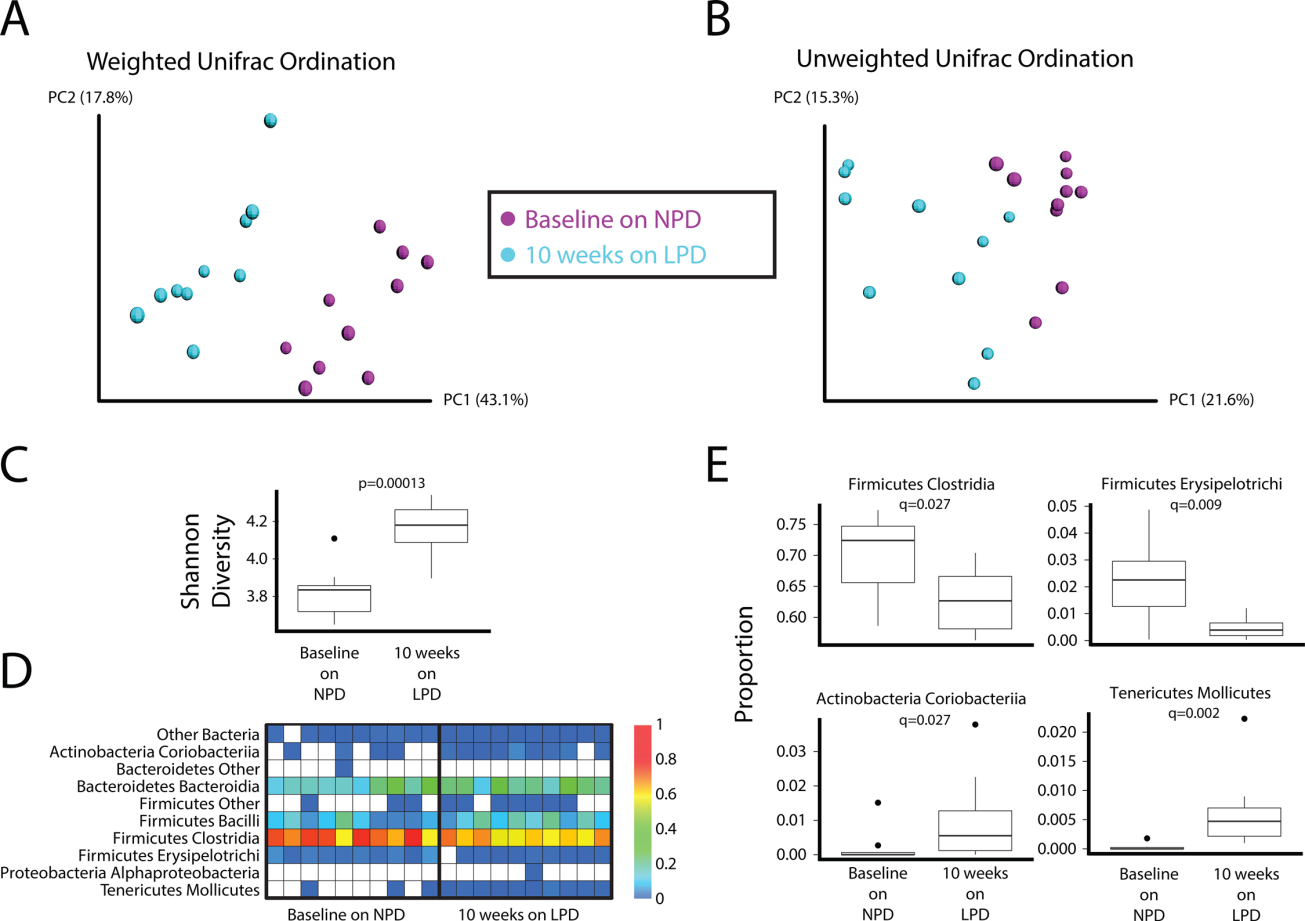
Effect of a LPD on the composition of the gut microbiota. Principal coordinates analysis (PCoA) ordination of mice before and after placement on the LPD for 10 weeks. Changes in community membership were analyzed using (A) weighted and (B) unweighted Unifrac. (C) The interquartile range of Shannon diversity values is shown for mice on the NPD who were later put on the LPD (Wilcoxon rank sum test p-value = 0.0001299). (D) Heatmap showing the relative abundance of bacterial lineages over time in mice who were on the NPD at baseline and then after ten weeks on the LPD. Rows indicate bacterial lineages annotated at the class taxonomic level on the left. The color key on the right of the figure indicates relative abundance. Columns summarize the sequencing results from individual fecal specimens. Each column represents a different mouse. The columns are grouped by diet. (E) Bacterial lineages that change on the LPD. Four bacterial classes significantly differed between the NPD and LPD (FDR-corrected Wilcoxon test p-value < 0.05). Relative abundance of each class in both diet groups is shown. Box and whiskers show the interquartile range; black circles mark the outlier samples.

16S tagged sequencing (1045 to 5305 reads per sample, median = 3448 reads) revealed modest yet distinct differences in the composition of the fecal microbiota in mice after placement on the LPD. These difference can be visualized in principal coordinates analysis of weighted (**Fig 2A**) and unweighted (**Fig 2B**) UniFrac distance. The LPD led to a significant increase in the diversity of the gut microbial community as assessed by the Shannon diversity index (**Fig 2C and S1 Fig**). The LPD also led to significant increases in the relative abundance of Mollicutes and Coriobacteria and a decrease in the Firmicutes classes Erysipelotrichi and Clostridia (**Fig 2D and 2E**).

### LPD has no effect on the initial colonization of ASF into the host microbiota

**Fig 3:**
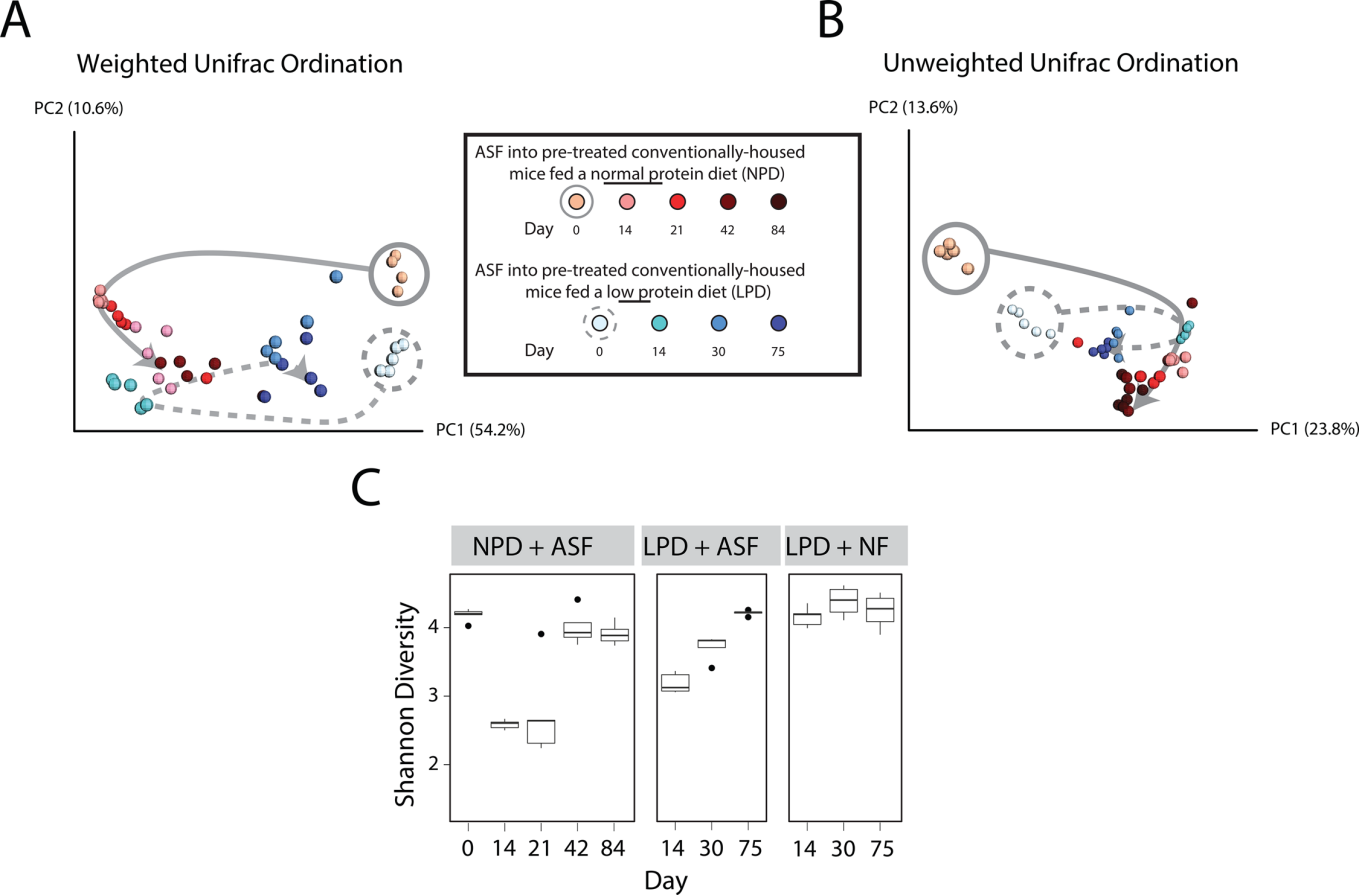
Effect of a LPD on the initial colonization of ASF and subsequent resilience over time. Principal coordinates analysis (PCoA) ordination of mice after transplantation with ASF. Changes in community membership were analyzed using (A) weighted and (B) unweighted Unifrac. Dietary groups are color coded as indicated with the shades of colors indicating progression in time. Day 0 samples have gray circles around them (solid for NPD, dashed for LPD). The arrows were added to help visualize the progression of time after ASF transplantation. (C) The interquartile range of Shannon diversity values are shown for mice on the NPD and LPD inoculated with either ASF or Normal Feces (NF). Black circles mark the outlier samples.

We have previously shown that there is a reduction in gut bacterial biomass upon treatment of mice with oral antibiotics (vancomycin and neomycin) and PEG, thus permitting the colonization of ASF upon inoculation by oral gavage [13]. However, the effect of a LPD on the colonization of ASF into the gut of a prepared host remains unknown. After preparation with antibiotics and PEG, we orally inoculated five of the LPD-fed mice with ASF (herein referred to as “ASF-transplanted”). As a control group, we transplanted the other five LPD-fed mice using feces from conventionally-reared donor mice (herein referred to “NF-transplanted,” for Normal Feces). Using 16S rRNA tagged sequencing, we tracked taxonomic alterations in the gut microbiota over time. We found that NF-transplanted mice exhibited minimal change in the composition of their gut microbiota (**S2 Fig**). However, the gut microbiota of ASF-transplanted mice underwent a shift in composition in a similar fashion to that previously observed in NPD-fed mice transplanted with ASF [13], as shown in **Fig 3A and 3B.** In particular, the shifts along PC1 in both cohorts of mice represent changes due to the initial ASF inoculum (compare days 0 to 14 in both groups), whereas differences between the two cohorts of mice along PC2 may represent the effect of diet. These findings suggest that a LPD does not affect the initial colonization of ASF into the host microbiota.

### Diet affects the resilience of the gut microbiota engineered by inoculation with ASF

**Fig 4:**
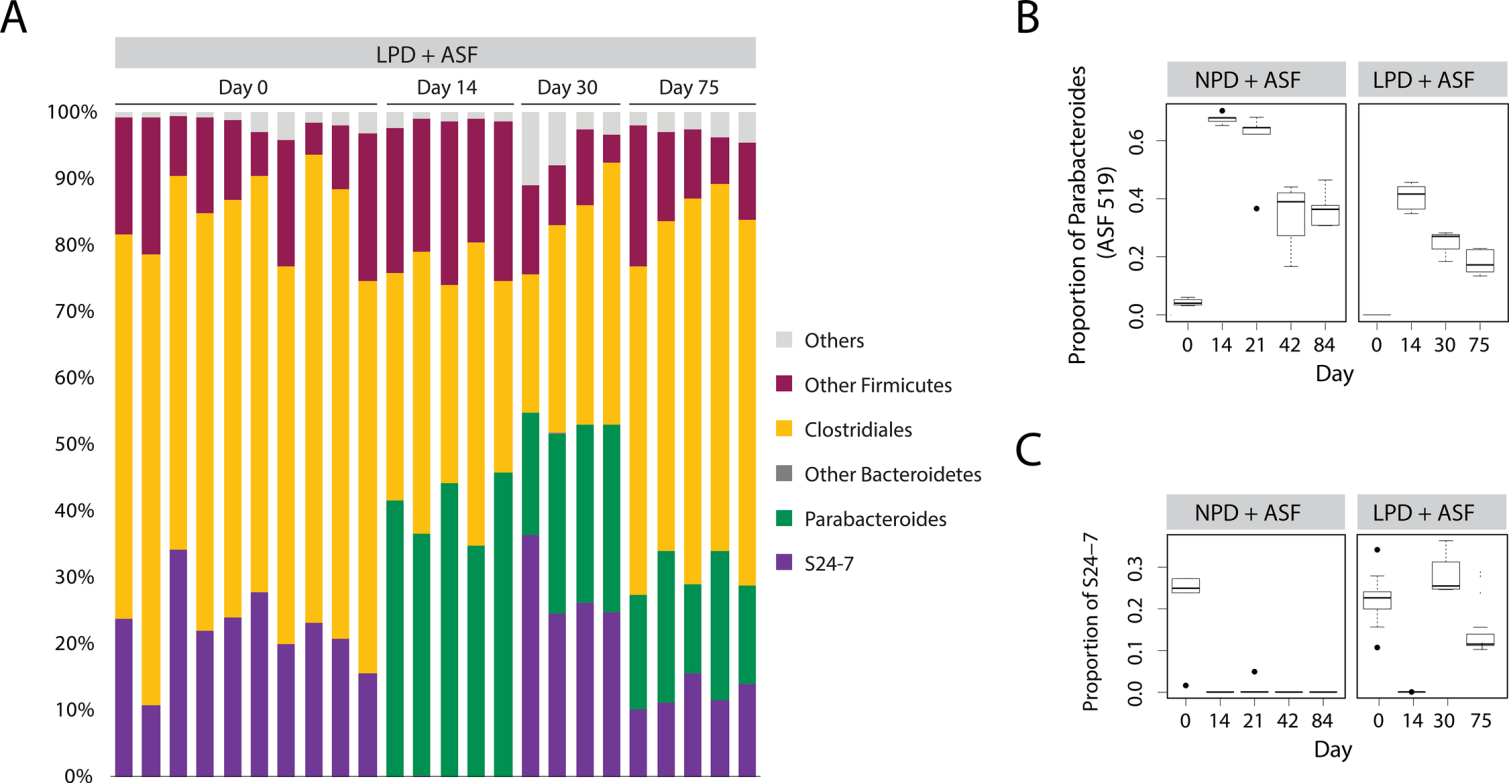
S24-7 returns after ASF transplantation into mice on a LPD but not on a NPD. (A) Relative abundance of bacterial taxa are shown. Each column represents a single sample of a pre-treated, ASF-transplanted mice on the LPD (LPD + ASF). Progression of inoculation is shown across multiple days post inoculation with ASF. Relative abundance of (B) Parabacteroides (including ASF 519) and (C) S24-7. Box and whiskers show the interquartile range; black circles mark the outlier samples.

By tracking the composition of mice inoculated with ASF, we determined the effect of a LPD on the ability of ASF to engineer a different microbiota composition. In the setting of a NPD, we previously observed that ASF transplantation led to the development of a new steady state community after one month composed of both ASF and the return of selected taxa of the Firmicutes phylum, but no non-ASF Bacteroidetes [13]. We proposed that *Parabacteroides* ASF519, the dominant taxon in ASF in feces, may have prevented the return of other Bacteroidetes taxa by competitive niche exclusion. Tracking compositional changes in the gut microbiota over time, we found that in the setting of the LPD, the gut microbiota engineered by ASF transplantation developed into an alternative rich community with diversity similar to that on the NPD (**Fig 3C**). However, ASF519 did not suppress the return of other Bacteroidetes. Instead, Bacteroidetes S24-7, a poorly classified yet common bacterial taxon in the commensal murine gut microbiota [25, 26], returned after ASF transplantation and reached an equilibrium state with ASF519 (**Fig 4 and S3 Fig**). We plotted the progression of the transplanted ASF community over time. We found that ASF reached a new steady state in the setting of the LPD at around 4 weeks after transplantation, similar to what we previously observed in the setting of the NPD [13] (**Fig 3A and 3B**). However, this steady state more closely resembled the endogenous microbiota, likely as a result of the return of S24-7 on the LPD (best observed in **Fig 3A** along PC1 –compare the solid to dotted grey line). Overall, these findings suggest that ability of ASF lineages to compete is reduced in the presence of a LPD.

### The ASF-engineered gut microbiota lowers fecal ammonia more effectively than LPD alone

**Fig 5:**
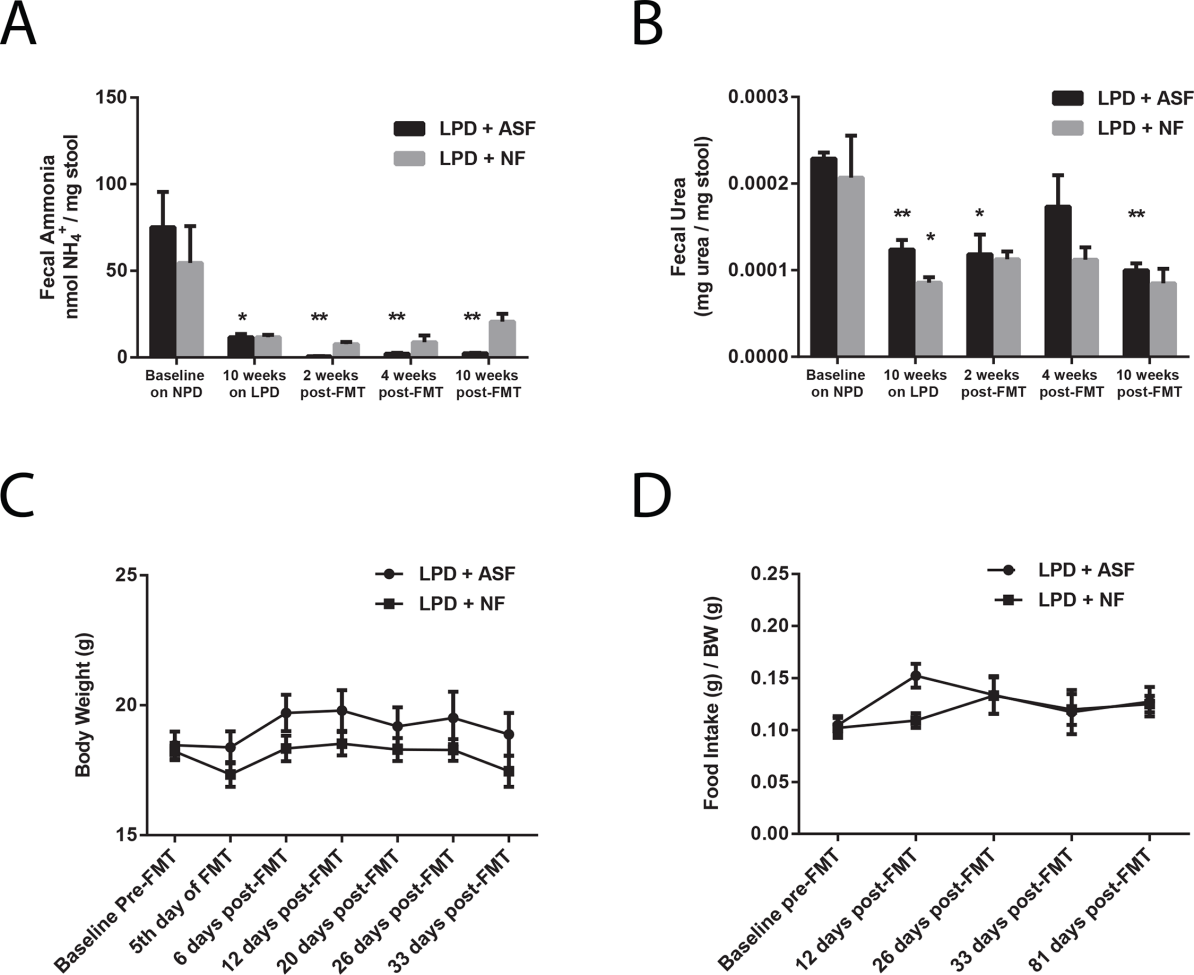
ASF transplantation alters colonic urea nitrogen recycling without significantly affecting host physiology. (A) ASF transplantation reduces fecal ammonia below the level achieved by the LPD alone (n = 4–5 per group, *p<0.05 compared to baseline, **p<0.01 compared to 10 weeks on the LPD). (B) No difference in fecal urea level between ASF- and NF-transplanted mice (n = 2–5 per group, *p<0.05 compared to baseline, **p<0.01 compared to baseline). No difference in (C) body weight or (D) food intake between ASF- and NF-transplanted mice (n = 5 per group). Values represent mean ± SEM. Significance determined by two-tailed Student’s t-test.

We have previously shown that ASF transplantation durably reduces fecal ammonia by decreasing fecal urease activity [13]. Since a LPD itself mainly reduces fecal ammonia by decreasing the delivery of urea to the colon (**Fig 1E and 1F**), we sought to determine whether the ASF-engineered microbiota would be able to reduce fecal ammonia levels below those achieved by a LPD alone. We measured fecal urea and fecal ammonia in mice at baseline on the NPD, after ten weeks on the LPD, and compared ASF and NF transplantation on the LPD. As shown in **Fig 5A,** after the initial reduction in fecal ammonia levels induced by the LPD, ASF transplantation reduced fecal ammonia further than did NF transplantation. The ability of the ASF-engineered microbiota to lower fecal ammonia levels below those achieved by the LPD alone is likely due to the reduction in fecal urease activity since there was no difference in fecal urea levels after NF and ASF transplantation (**Fig 5B**). These results indicate that the functionality of the ASF-engineered gut microbiota is not significantly altered in the setting of a LPD despite alterations in its composition.

### The low fecal urease and fecal ammonia-producing microbiota engineered by ASF inoculation does not exacerbate host metabolic dysfunction induced by LPD

Urea is a nitrogenous waste product, but it is thought to contribute to host nutrition via urea nitrogen recycling by intestinal bacterial urease in both ruminants and non-ruminants, leading to microbial and/or host synthesis of peptides, amino acids, and other small molecules [17]. We asked whether this role of urea may become important for host physiology in the setting of a LPD, where systemic nitrogen is reduced. After transplanting the above cohorts of mice with ASF or NF, we continued to monitor their body weight, food intake, and survival. Remarkably, despite the absence of colonic urea nitrogen recycling, ASF-transplanted mice did not differ significantly from NF-transplanted mice (**Fig 5C and 5D and S4 Fig**). Thus, in the setting of a LPD, ASF transplantation does not lead to significant detrimental changes to host physiology and metabolism.

## Discussion

The success of FMT in the treatment of recurrent *Clostridium difficile* infections provides proof of concept that the gut microbiota can be a target for the treatment of disease in humans. The use of fecal transfer will likely be replaced by the use of defined microbial consortia with specific biological properties. As proof of concept, we have shown in murine models that a defined consortium of eight bacteria, known as Altered Schaedler Flora (ASF), can be used to engineer the gut microbiota with altered functionality, namely a reduction in fecal urease activity and ammonia production [13]. Critical to the success of this strategy is the substantial reduction in the biomass of the baseline microbiota to provide a niche into which the bacterial inoculum can colonize.

An important consideration for engineering the gut microbiota is resilience to environmental stress. An optimally engineered microbiota would be a rich community that stays intact in the presence of environmental stress. Diet is an important environmental stressor on the gut microbiota that should be considered when engineering gut microbial communities. As one example, the low ammonia-producing microbiota engineered by ASF, which has functional durability for several months in mice fed an irradiated diet, shows reduced resilience when the mice are fed a non-irradiated diet [13]. We chose to study the impact of dietary protein on the resilience of the ASF-engineered microbiota for several reasons: 1) Dietary protein has been shown to influence the composition of the gut microbiota in gnotobiotic mice [27]; 2) Protein consumption regulates the production of hepatic urea that may affect colonic urea delivery to the gut microbiota [17, 28]; 3) Protein-restricted diets are an important therapeutic modality for patients with hyperammonemic inborn errors of metabolism [23, 29].

Unlike the modulation of fat and fiber in mice, which have been shown to have a strong effect on the composition of the murine gut microbiota [1, 27], we show that severe restriction of dietary protein had a modest effect. Within the Firmicutes phylum the Clostridia and Erysipelotrichi classes decreased significantly on a LPD, consistent with the preference of taxa within Firmicutes, particularly Clostridia species, to metabolize amino acids and peptides [30, 31]. Alternatively, since we show that a LPD reduces serum urea concentrations with reduced delivery to the colon resulting in lower fecal ammonia levels, an alteration in nitrogen flux via ammonia into the gut microbiota [32, 33] may also have an effect on the composition of the bacterial microbiota.

Since we balanced protein with carbohydrate in the composition of the purified rodent diets, it is difficult to ascertain if the differences in the composition of the gut microbiota are due primarily to alterations in protein or in carbohydrate. We found that two bacterial phyla present at low abundances increased significantly on a LPD. Specifically, the classes Mollicutes (Tenericutes phylum) and Coriobacteria (Actinobacteria phylum) increased on a LPD. Previous work has shown that Mollicutes proliferated on a typical Western diet characterized by high-fat/high-sugar content, likely because of their ability to import and process simple sugars [34]. Thus, an increase in the abundance of Mollicutes that we observed on a LPD could be due to the increase in carbohydrate content rather than the reduction in protein content. Another study also showed that gut colonization by Actinobacteria and Tenericutes was strongly correlated with decreased hepatic levels of glycogen and glucose [35], further suggesting the interplay between the host and these two phyla may be closely related to carbohydrate metabolism.

Despite the effect of a LPD on the composition of the murine gut microbiota at baseline, this did not have an effect on the initial colonization of ASF at 2 weeks, demonstrating that the use of antibiotics and PEG effectively prepared the environment of the gut for inoculation by a minimal defined bacterial consortium. Subsequently, development of the resultant engineered microbiota, determined by emergence of various bacterial taxa in addition to ASF, was distinctly different in mice fed a NPD versus a LPD. On a NPD, we previously showed that the dominant taxon *Parabacteroides* (ASF519) was able to exclude the entire Bacteroidetes phylum yet permit the reappearance of specific taxa belonging to the Firmicutes phylum. The observation that bacterial lineages with similar phylogeny exhibit competitive niche exclusion has been demonstrated in the *Bacteroides* genus where successful competition for carbohydrate substrates plays an important role [36]. By contrast, on a LPD, the resultant engineered microbiota appears to be more similar to baseline primarily due to the reemergence of a single bacterial taxon belonging to the Bacteroidetes family, S24-7.

S24-7 has been previously recognized as a dominant taxonomic group in the murine microbiota. It was first characterized by Salzman *et al*., who referred to the taxon as “mouse intestinal bacteria” [25]. The S24-7 taxon is phylogenetically distinct from other named genera in the order Bacteroidales. The taxon has been reported as altered in several recent mouse studies: it was increased in proportion following partial hepatectomy [37], associated with co-infection by *Hymenolepis* spp. [38], and decreased in proportion following antibiotic treatment for parenteral nutrition-associated liver injury [39]. However, to our knowledge, no study has previously characterized competition between S24-7 and other Bacteroidetes species in mice. The S24-7 taxon is typically encountered at very low (<1%) abundance in fecal samples from human populations. However, one study of a previously uncontacted Amerindian population reported the taxon to be enriched in isolated Yanomami Amerindians relative to Guahibo Amerindian, Malawian, and U.S. subjects [40]. The average abundance of the taxon in Yanomami Amerindians was reported to be nearly 5% of total bacteria, suggesting a potential role for S24-7 in the human gut.

Upon reduction of bacterial biomass through a combination of antibiotics and PEG, S24-7 is no longer detectable and shows no return over time after mice have been inoculated with ASF. Since both *Paracteroides* (ASF519) and S24-7 are closely related within the Bacteroidetes phylum, we speculate that S24-7 may be co-excluded from the luminal gut environment by ASF519 through competitive niche exclusion, a mechanism that has been hypothesized as the basis for the inversely-related proportions of Bacteroides and Prevotella in the human gut microbiota [2, 41], a predominant feature of “enterotypes” [42]. From a mechanistic standpoint, the basis of competitive niche exclusion may involve the competition of metabolic substrates as has been demonstrated for Bacteroides species in a reductionist model system [36]. Since S24-7 reappears and co-exists at approximately equal levels with ASF519 in LPD-fed mice, the alteration of substrate availability via diet may have altered the luminal environment of the gut that reduces the need for competition between these two taxa. For example, a LPD may have altered the balance of nitrogen flux into the gut microbiota via the uptake of ammonia. Indeed, despite the return of S24-7 and the similarities between the composition of the gut microbiota of a conventionally-housed mouse and the ASF-engineered community established in LPD-fed mice, fecal ammonia levels remained much lower in ASF-transplanted mice than those transplanted with normal feces. This suggests that S24-7 may be urease negative. Further elucidation of such mechanism(s) will require genomic characterization of S24-7 along with an evaluation of its biological properties.

The quantification of fecal ammonia was used to determine the impact of microbiota composition on the function of the community. Despite the modest alterations in the gut microbiota induced by the consumption of a LPD, there was a significant reduction in fecal ammonia levels reflecting the reduced abundance of urea substrate available for hydrolysis by the gut microbiota. This observation emphasizes the notion that diet may have an indirect impact on the gut microbiota by alteration of the host similar to the outgrowth of a pathobiont due to the enhanced production of sulfated bile acids in mice fed milk fat [43]. Importantly, engineering of the gut microbiota using ASF led to a reduction in fecal ammonia levels significantly greater than that observed on a LPD.

Since ammonia, produced by the gut microbiota via urease activity, is absorbed by the host where it can be used for amino acid synthesis, it has been hypothesized that this form of nitrogen recycling may be important for host health especially under conditions of limited protein intake [17, 44]. This might be a significant limitation of a strategy focused on reducing gut microbiota ammonia production for the treatment of hyperammonemia and hepatic encephalopathy [13]. Although LPD-fed mice did not exhibit growth, as would be expected, ASF transplantation with subsequent robust reduction of fecal ammonia levels did not lead to any effects on food intake, growth, or mortality relative to LPD-fed mice transplanted with normal feces who had much higher levels of fecal ammonia. Since patients with hyperammonemic inborn errors of metabolism are placed on a LPD to prevent metabolic crises, our observations provide preliminary evidence that the engineering of gut microbiota to reduce fecal ammonia production may be well tolerated in this patient population. However, additional safety studies are needed.

In summary, we show that diet has a significant effect on the ability of a defined microbial consortium to engineer the composition of the gut microbiota. Specifically, LPD alters the co-exclusion of two dominant taxa within the Bacteroidetes phylum. Given the alterations in the syntropic host-microbiota interactions in nitrogen flux that occur in the levels of urea delivery from the host to the gut microbiota, the reduced production of ammonia via bacterial urease, and the uptake of ammonia by both the host and the gut microbiota, a LPD may be a particularly important environmental stressor that will impact upon the composition of an engineered microbiota. Nevertheless, the functionality of the engineered gut microbiota, as quantified by a reduction in fecal ammonia levels, remained intact. Together with the absence of detrimental effects on host physiology in the setting of a LPD, the reduction in fecal ammonia levels via engineering of the gut microbiota may be an effective therapeutic strategy for patients with hyperammonemic inborn errors of metabolism.

## Materials and Methods

### Animals

C57B6J female mice 8 to 12 weeks old (The Jackson Laboratory) were used in this study. Fecal pellets collected from five ASF-colonized CB17 SCID mice (Taconic) served as the source of the ASF inoculum whereas five conventionally-colonized C57B6J mice (The Jackson Laboratory) served as the source of the normal feces (NF) inoculum used in the FMT procedures as previously described [13]. Fecal homogenates were prepared by diluting 0.1 g feces 10-fold in PBS. Mice were prepared for FMT by oral delivery of antibiotics in drinking water (1.125 g aspartame, 0.15 g vancomycin, and 0.3 g neomycin in 300 mL sterile water) for 72 hours. During the final 12 hours, the water supply was exchanged with a 10% PEG solution (Merck), and the mice were fasted. Mice were then inoculated daily with fecal homogenates by oral gavage for 7 days. All mice were housed five per cage in a conventional specific-pathogen free (SPF) facility (and transferred from one conventional facility to another conventional facility within the University of Pennsylvania 10 weeks after the start of experiment for NMR imaging) and fed irradiated AIN-76A (Research Diets D10001, 21% protein by kilocalories–NPD, see **Table 1**). After one week, ten mice were switched to irradiated AIN-76A with lower protein content (Research Diets D08092201, 3% protein by kilocalories–LPD, see **Table 1**). Fecal pellets were collected at baseline on NPD, 10 weeks after placement on LPD, 2 weeks after FMT (15 weeks on LPD), 4 weeks after FMT (17 weeks on LPD), and 10 weeks after FMT (23 weeks on LPD) for bacterial taxonomic and biochemical analyses. Fecal pellets were collected in 1.5 mL microcentrifuge tubes (Sigma-Aldrich) and immediately placed on dry ice then stored in -80°C freezer until time of analysis. Body composition was determined after ten weeks on respective diets using NMR imaging via the Mouse Phenotyping, Physiology and Metabolism Core at the University of Pennsylvania. One mouse in the NF FMT group died of natural cause on Day 120 of experiment (approximately one month after FMT completion). No other mice exhibited symptoms indicative of severe illness or moribundity requiring medical treatment or euthanasia. All animal studies were performed with the approval of the Institutional Animal Care and Use Committee of the University of Pennsylvania (Protocol Number: 803408).

### 16S V1-V2 Sequencing

DNA was isolated from stool as previously described [2, 45]. 100 ng of DNA was amplified with barcoded primers annealing to the V1-V2 region of the 16S rRNA gene (forward primer, 5′-AGAGTTTGATCCTGGCTCAG-3′; reverse primer, 5′-CTGCTGCCTYCCGTA-3′; [46, 47] using AccuPrime Taq DNA Polymerase System with Buffer 2 (Life Technologies). PCR reactions were performed on a thermocycler using the following conditions: initiation at 95°C for 5 min followed by 20 cycles of 95°C × 30 s, 56°C × 30 s, and 72°C × 1 min 30 s, then a final extension step at 72°C for 8 min. The amplicons from each DNA sample, which was amplified in quadruplicate, were pooled and purified with Agencourt AMPure XP beads (Beckman Coulter) following the manufacturer’s instructions. Purified DNA samples were then sequenced using the 454/Roche GS FLX Titanium chemistry (454 Life Sciences).

### 16S rRNA Gene Sequence Analysis

16S rRNA gene sequence data was processed with QIIME v 1.8.0 [48] using default parameters. Sequences were clustered into operational taxonomic units (OTUs) at 97% similarity and then assigned Greengenes taxonomy [49] using the uclust consensus taxonomy classifier. Sequences were aligned using PyNAST [50] and a phylogenetic tree was constructed using FastTree [51]. Weighted and unweighted UniFrac [52] distances were calculated for each pair of samples for assessment of community similarity and generation of principal coordinate analysis (PCoA) plots. Statistical analyses for bacterial abundance difference was performed using non-parametric Wilcoxon test, and p-values were corrected for multiple comparisons using the Benjamini and Hochberg procedure.

### Measurement of fecal ammonia

Fecal ammonia concentrations were determined using an Ammonia Assay Kit (ab83360, Abcam, Cambridge, MA). Fecal pellets were suspended in the assay buffer provided at a concentration of 1 mg/10 uL, homogenized, and centrifuged at 13,000 x g for 10 minutes at room temperature to remove insoluble material. Ammonia concentration was then determined according to the kit protocol.

### Measurement of serum and fecal urea

Urea concentrations were determined using the QuantiChrom™ Urea Assay Kit (DIUR-500, Bioassay Systems, Hayward, CA). Serum samples were assayed directly. Fecal pellets were suspended in ddH_2_O at a concentration of 1 mg/10uL, homogenized, and centrifuged at 2,500 x g for 10 minutes at room temperature to remove insoluble material. Urea concentration was then determined according to the kit protocol.

## Supporting Information

S1 Fig Diversity in each mouse after a LPD. Shannon diversity is shown for all ten mice while on the NPD (blue) and after ten weeks on the LPD (salmon).
(EPS)

S2 Fig Principal coordinates analysis ordination of mice on the LPD after transplantation with ASF or NF. Changes in community membership were analyzed using (A) weighted and (B) unweighted Unifrac.
(EPS)

S3 Fig Relative abundance of bacterial taxa after ASF transplantation. Each bar represents a single sample. Samples represent pre-treated ASF-inoculated mice on the NPD (NPD + ASF). Progression is shown across multiple days post-inoculation with ASF.
(EPS)

S4 Fig Murine mortality on a LPD. Kaplan Meier curve showing no significant difference in survival between ASF- and NF-transplanted mice on the LPD (n = 5 per group at start of experiment).
(TIF)

S1 Table ASF consortium membership. ASF consortium number designation is indicated in the left column, and bacterial taxonomy at the genus or species level is indicated in the right column.
(DOCX)
