# Supplementary material for: Dietary Regulation of the Gut Microbiota Engineered by a Minimal Defined Bacterial Consortium
Source: PLoS One. 2016 May 13;11(5):e0155620. doi: 10.1371/journal.pone.0155620 (PMC4866709; doi:10.1371/journal.pone.0155620)
Supplement: S1 Table — ASF consortium number designation is indicated in the left column, and bacterial taxonomy at the genus or species level is indicated in the right column. (DOCX) [file pone.0155620.s005.docx]

| **ASF no.** | **Taxonomy** |
| --- | --- |
| ASF356 | *Clostridium* sp. |
| ASF360 | *Lactobacillus intestinalis* |
| ASF361 | *Lactobacillus murinus* |
| ASF457 | *Mucispirillum schaedleri* |
| ASF492 | *Eubacterium plexicaudatum* |
| ASF500 | *Pseudoflavonifactor* sp. |
| ASF502 | *Clostridium* sp. |
| ASF519 | *Parabacteroides goldsteinii* |
